# genozip: a fast and efficient compression tool for VCF files

**DOI:** 10.1093/bioinformatics/btaa290

**Published:** 2020-05-14

**Authors:** Divon Lan, Raymond Tobler, Yassine Souilmi, Bastien Llamas

**Affiliations:** School of Biological Sciences, The Environment Institute, Faculty of Sciences, The University of Adelaide, Adelaide, SA 5005, Australia

## Abstract

**Motivation:**

genozip is a new lossless compression tool for Variant Call Format (VCF) files. By applying field-specific algorithms and fully utilizing the available computational hardware, genozip achieves the highest compression ratios amongst existing lossless compression tools known to the authors, at speeds comparable with the fastest multi-threaded compressors.

**Availability and implementation:**

genozip is freely available to non-commercial users. It can be installed via conda-forge, Docker Hub, or downloaded from github.com/divonlan/genozip.

**Supplementary information:**

Supplementary data are available at *Bioinformatics* online.

## 1 Introduction

Large genomic projects are becoming increasingly common, resulting in Variant Call Format (VCF; Danecek *et al.*, 2011) files comprising thousands of individual genomic datasets. Even in their compressed form, such files are very large (typically several GB), rapidly driving up the cost of long-term data storage and file transfer and spurring the development of more efficient compression algorithms.

While a handful of new compression algorithms have recently emerged that work by compressing genotypes within VCF files (e.g. Deorowicz and Danek, 2019; Durbin, 2014; Kelleher *et al.*, 2019), genotypes are only one data type represented in a VCF file, and are often only a minor contributor to the total data content. For example, in the file used as the real-world example in (Durbin, 2014)—File1 in our benchmarks—the genotypes represent only 7.1% of the uncompressed VCF file data. Thus, it is clear that just compressing the genotypes is not sufficient as a compression strategy for VCF files.

Here, we present genozip, a lossless compression tool that greatly improves genomic data compression by utilizing algorithms specific to the data types common to VCF files. genozip can handle VCF files of any ploidy, phasing structure or variant type with up to 99 alternate alleles per variant, along with any FORMAT and INFO data. While the primary objective of genozip is optimal packaging of genomic data for efficient and secure storage and distribution, it also includes capabilities for pipeline analyses.

## 2 Software description

The genozip package runs on all popular operating systems and includes four command line tools—genozip, genounzip, genocat and genols. genozip receives one or more .vcf, .vcf.gz, .vcf.bz2, .vcf.xz or .bcf files or urls (FTP or HTTP) as input, and outputs one or more .vcf.genozip files, while genounzip decompresses .vcf.genozip files back to .vcf or .vcf.gz format and genols provides statistics regarding the contents of .genozip files.

To support seamless integration into analytical pipelines, the genocat command is provided to access data within .vcf.genozip files, and includes options like –regions and –samples that allow random access to data. Indexing is done as part of the compression and there is no separate indexing step or index file. In addition, the toolset is designed to enable use of standard input/output streams.

By encrypting the data with –password (using 256 bit AES), genozip enables efficient and secure distribution of genomic files that comply with stringent privacy requirements. Data integrity is further ensured by generating an MD5 signature with –md5. Additionally, the -output option concatenates VCF files with identical samples and the original components can be regenerated using –split.

We have included several additional options that allow the user to optimize compression according to their needs. First, the –optimize option improves compression by modifying data in some INFO and FORMAT subfields by rounding floating point numbers to 2 significant digits and capping Phred values. Note that in this case the VCF data are modified, and therefore the compression is not lossless, but this does not impact downstream analytical results. Second, the –gtshark option makes use of the GTShark algorithm (Deorowicz and Danek, 2019) that improves compression ratios compared to using either genozip or GTShark alone (see Supplementary Material). Finally, the –vblock and –sblock options allow the user to control the tradeoff between compression and speed related to subsetting regions and samples.

Note that some options require the appropriate tools to be installed: compressing .bcf files into .genozip format requires bcftools, compressing .xz files requires XZ Utils (Collin, 2011), decompressing into .vcf.gz requires bgzip, using –gtshark requires GTShark, and compressing from an URL requires cURL (Hostetter *et al.*, 1997).

## 3 Benchmark

To evaluate genozip’s performance, we compared its compression ratios and speeds on two different VCF files from The 1000 Genomes Project Consortium (2012)—‘File1' which is rich in FORMAT subfields and 'File2' which is rich in genotype data (see Supplementary Table S1)—against a wide range of tools. All benchmarks were conducted on the same machine that has 56 physical cores (4 X Intel^®^ Xeon^®^ Gold 6132 CPU @ 2.60 GHz) and 755 GB of usable memory. More details, including benchmarks against genotype compression tools such as BGT (Li, 2016) and GTC (Danek and Deorowicz, 2018) that are not capable of compressing arbitrary VCF files losslessly are available in Supplementary Material.

**Fig. 1.**
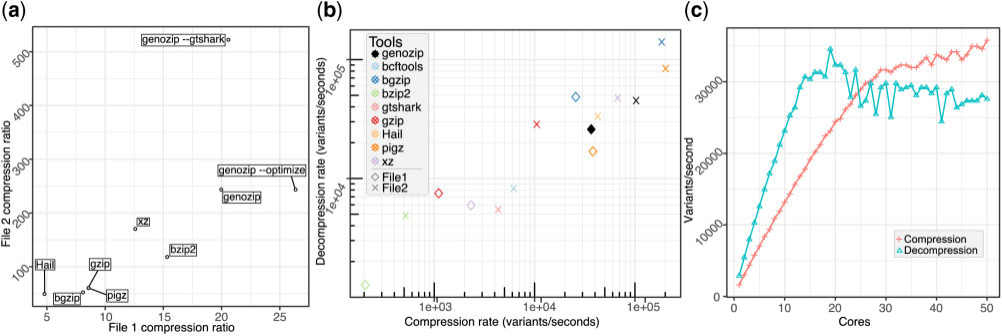
Benchmarking genozip performance. (**a**) Compression ratios for genozip using three different options relative to five other commonly used compression tools (see labels) for two VCF files, the FORMAT-subfields-rich data (*x*-axis) and genotype-rich data dominant (*y*-axis). (**b**) Compression (*x*-axis) and decompression (*y*-axis) rates for genozip and five other tools on the two VCF files (see inset key), and the rates (**c**) genozip execution scalability with used CPU cores (see Supplementary Material)

For both tested VCF files, the compression ratios achieved by genozip are considerably higher than other tested tools (Fig. 1a). Further, genozip offers one of the fastest compression/decompression speeds amongst the tested tools (Fig. 1b), indicating that performance gains are achieved without negatively impacting run times. To achieve high processing speeds, genozip implements an advanced memory and thread management strategy that scales across 10 s of cores (Fig. 1c).

## 4 Conclusion

genozip is a user friendly and fully featured compression software that readily integrates into any standard bioinformatics pipeline. genozip achieves compression ratios significantly better than other standard tools, by exploiting redundancies in the data that are specific to biological data and that are not evident by textual analysis alone. Moreover, genozip achieves significant gains to compression speed relative to other tools by taking full advantage of modern computational hardware, including multi-core processors and multi-gigabyte RAM, whenever available. By default, genozip dynamically balances its internal execution pipelines to maximize utilization of all the available resources.

## Supplementary Material

btaa290_Supplementary_Data
