# Supplementary material for: genozip: a fast and efficient compression tool for VCF files
Source: Bioinformatics. 2020 May 14;36(13):4091–2. doi: 10.1093/bioinformatics/btaa290 (PMC7332572; doi:10.1093/bioinformatics/btaa290)
Supplement: btaa290_Supplementary_Data [file btaa290_supplementary_data.pdf]

Supplementary Information

for

genozip: a fast and efficient compression tool for VCF files

Divon Lan[1]*, Ray Tobler[1], Yassine Souilmi[1 †], Bastien Llamas[1 † *]

[1] School of Biological Sciences, The Environment Institute, Faculty of Sciences, The University of Adelaide, Adelaide SA 5005, Australia

[†] Equal contribution

* Corresponding authors: DL (divon.lan@adelaide.edu.au) and BL (bastien.llamas@adelaide.edu.au)

Content

1. Full list of options of **`genozip, genounzip, genocat`** and **`genols`**
2. Implementation
3. Compression ratio and speed benchmarks in more detail
4. Benchmarking of genotype-only compression algorithms
5. Core scalability test - raw data

1. Full list of options of genozip, genounzip, genocat and genols

Compress VCF (Variant Call Format) files

```
Usage: genozip [options]... [files or urls]...

See also: genounzip genocat genols

Supported input file types: .vcf .vcf.gz .vcf.bgz .vcf.bz2 .vcf
                     .xz .bcf .bcf.gz .bcf.bgz
Note: for .bcf files, bcftools needs to be installed, and for
                     .xz files, xz needs to be installed

Examples: genozip file1.vcf file2.vcf -o concat.vcf.genozip
          genozip --optimize --password 12345 ftp://ftp.ncbi.nlm
                     .nih.gov/file2.vcf.gz

Actions - use at most one of these actions:
   -d --decompress   Same as running genounzip. For more details,
                     run: genounzip --help

   -l --list         Same as running genols. For more details,
                     run: genols --help

   -h --help         Show this help page. Use with -f to see
                     developer options.

   -L --license      Show the license terms and conditions for
                     this product

   -V --version      Display version number

Flags:
   -c --stdout       Send output to standard output instead of a
                     file

   -f --force        Force overwrite of the output file, or
                     force writing .vcf.genozip data to standard
                     output

   -^ --replace      Replace the source file with the result
                     file, rather than leaving it unchanged

   -o --output       <output-filename>. This option can also be
                     used to concatenate multiple input files
                     with the same individuals, into a single
```

concatenated output file

-p --password <password>. Password-protected - encrypted with 256-bit AES

-m --md5 Calculate the MD5 hash of the VCF file. When the resulting file is decompressed, this MD5 will be compared to the MD5 of the decompressed VCF.
Note: for compressed files, e.g. myfile.vcf.gz, the MD5 calculated is that of the original, uncompressed file.

-q --quiet Do not show the progress indicator or warnings

-Q --noisy Stop the suppression of warnings

-t --test After compressing normally, decompress in memory (i.e. without writing the decompressed file to disk) - comparing the MD5 of the resulting decompressed file to that of the original VCF. This option also activates --md5.

-@ --threads <number>. Specify the maximum number of threads. By default, this is set to the number of cores available. The number of threads actually used may be less, if sufficient to balance CPU and I/O.
Tip: if you're concerned about sharing the computer with other users, rather than using --threads to reduce the number of threads, a better option would be to use the command nice, e.g. 'nice genozip....'. This yields CPU to other users if needed, but still uses all the cores that are available

--show-content Show the information content of VCF files and the compression ratios of each component

Optimizing:

-9 --optimize Modify the VCF file in ways that are likely insignificant for analytical purposes, but

make a significant difference for compression. At the moment, these optimizations include:
- PL data: Phred values of over 60 are changed to 60.   Example: '0,18,270' -> '0,18,60'
- GL data: Numbers are rounded to 2 significant digits.   Example: '-2.61618,-0.447624,-0.193264' -> '-2.6,-0.45,-0.19'
- GP data: Numbers are rounded to 2 significant digits, as with GL.
- VQSLOD data: Number is rounded to 2 significant digits. Example: '-4.19494' -> '-4.2'

Note: due to these data modifications, files compressed with --optimized are NOT identical as the original VCF after decompression. For this reason, it is not possible to use this option in combination with --test or --md5

-B --vblock <number between 1 and 2048>. Set the maximum size of memory (in megabytes) of VCF file data that can go into one variant block. By default, this is set to 128 MB. The variant block is the basic unit of data on which genozip and genounzip operate. This value affects a number of things: 1. Memory consumption of both compression and decompression are linear with the variant block size. 2. Compression is sometimes better with larger block sizes, in particular if the number of samples is small. 3. Smaller blocks will result in faster 'genocat --regions' lookups

-S --sblock <number>. Set the number of samples per sample block. By default, it is set to 4096. When compressing or decompressing a variant block, the samples within the block are divided to sample blocks which are compressed separately. A higher value will result in a better compression ratio, while a lower value will result in faster 'genocat --samples' lookups

```
--gtshark          Use gtshark instead of the default bzlib as
                   the final compression step for allele data
                   (the GT subfield in the sample data).
                   Note: For this to work, gtshark needs to be
                   installed - it is a separate software
                   package that is not affiliated with genozip
                   in any way. It can be found here: https:/
                   /github.com/refresh-bio/GTShark.
                   Note: gtshark also needs to be installed
                   for decompressing files that were
                   compressed with this option.
```

One or more file names may be given, or if omitted, standard input is used instead

Uncompress VCF (Variant Call Format) files previously compressed with genozip

Usage: **genounzip** [options]... [files]...

See also: genozip genocat genols

```
Examples: genounzip file1.vcf.genozip file2.vcf.genozip
          genounzip file.vcf.genozip --output file.vcf.gz
          genounzip concat.vcf.genozip --split
```

Options:

-c --stdout	Send output to standard output instead of a file
-z --bgzip	Compress the output VCF file(s) with bgzip. Note: this option is implicit if --output specifies a filename ending with .gz or .bgz. Note: bgzip needs to be installed for this option to work
-f --force	Force overwrite of the output file
-^ --replace	Replace the source file with the result file, rather than leaving it unchanged
-O --split	Split a concatenated file back to its original components
-o --output	<output-filename>. Output to this filename instead of the default one
-p --password	<password>. Provide password to access file (s) that were compressed with --password
-m --md5	Shows the MD5 hash of the decompressed VCF file. If the file was originally compressed with --md5, it also verifies that the MD5 of the original VCF file is identical to the MD5 of the decompressed VCF. Note: for compressed files, e.g. myfile.vcf.gz, the MD5 calculated is that of the original, uncompressed file.
-q --quiet	Do not show the progress indicator or warnings

```
   -Q --noisy        Stop the suppression of warnings

   -t --test         Decompress in memory (i.e. without writing
                     the decompressed file to disk) - comparing
                     the MD5 of the resulting decompressed file
                     to that of the original VCF. Works only if
                     the file was compressed with --md5

   -@ --threads      <number>. Specify the maximum number of
                     threads. By default, this is set to the
                     number of cores available. The number of
                     threads actually used may be less, if
                     sufficient to balance CPU and I/O.
                     Tip: if you are concerned about sharing the
                     computer with other users, rather than
                     using --threads to reduce the number of
                     threads, a better option would be to use
                     the command nice, e.g. 'nice genozip....'.
                     This yields CPU to other users if needed,
                     but still uses all the cores that are
                     available

   -h --help         Show this help page. Use with -f to see
                     developer options.

   -L --license      Show the license terms and conditions for
                     this product

   -V --version      Display version number

One or more file names must be given
```

Print VCF (Variant Call Format) file(s) previously compressed with genozip

Usage: **genocat** [options]... [files]...

See also: genozip genounzip genols

```
Options:
   -r --regions      [^]chr|chr:pos|pos|chr:from-to|chr:from-
                     |chr:-to|from-to|from-|-to[,...]
                     Show one or more regions of the file.
                     Examples:
                               genocat myfile.vcf.genozip -r22
                     :1000000-2000000  (A range of chromosome 22)
                               genocat myfile.vcf.genozip -r
                     -2000000,2500000-   (Two ranges of all
                     chromosomes)
                               genocat myfile.vcf.genozip -r21
                     ,22               (All of chromosome 21 and
                     22)
                               genocat myfile.vcf.genozip -r^MT
                     ,Y               (All of chromosomes except
                     for MT and Y)
                               genocat myfile.vcf.genozip -r^
                     -10000             (All sites on all
                     chromosomes, except positions up to 10000)
                     Note: genozip files are indexed
                     automatically during compression. There is
                     no separate indexing step or separate index
                     file.
                     Note: Indels are considered part of a
                     region if their start position is.
                     Note: Multiple -r arguments may be
                     specified - this is equivalent to chaining
                     their regions with a comma separator in a
                     single argument

   -t --targets      Identical to --regions, provided for
                     pipeline compatibility

   -s --samples      [^]sample[,...]
                     Show a subset of samples (individuals).
                     Examples:
                               genocat myfile.vcf.genozip -s
                     HG00255,HG00256    (show two samples)
                               genocat myfile.vcf.genozip -s
```

```
                  ^HG00255,HG00256   (show all samples except
                  these two)
                  Note: This does not change the INFO data
                  (including the AC and AN tags).
                  Note: sample names are case-sensitive.
                  Note: Multiple -s arguments may be
                  specified - this is equivalent to chaining
                  their samples with a comma separator in a
                  single argument

-G --drop-genotypes Output the data without the individual
                  genotypes and FORMAT column

-H --no-header    Do not output the VCF header

   --header-only  Output only the VCF header

   --GT-only      For samples, output only genotype (GT) data,
                  dropping the other subfields

   --strip        Do not output values for ID, QUAL, FILTER,
                  INFO; FORMAT is only GT (at most); Samples
                  include allele values (i.e. GT subfield)
                  only

-o --output       <output-filename>. Output to this filename
                  instead of stdout

-p --password     Provide password to access file(s) that
                  were compressed with --password

-@ --threads      Specify the maximum number of threads. By
                  default, this is set to the number of cores
                  available. The number of threads actually
                  used may be less, if sufficient to balance
                  CPU and I/O.
                  Tip: if you're concerned about sharing the
                  computer with other users, rather than
                  using --threads to reduce the number of
                  threads, a better option would be to use
                  the command nice, e.g. 'nice genozip....'.
                  This yields CPU to other users if needed,
                  but still uses all the cores that are
                  available

-q --quiet        Do not show warnings
```

```
   -Q --noisy          Stop the suppression of warnings
   -h --help           Show this help page. Use with -f to see
                       developer options. Use --header-only if
                       that is what you are looking for

   -L --license        Show the license terms and conditions for
                       this product

   -V --version        Display version number

One or more file names must be given
```

View metadata of VCF (Variant Call Format) files previously compressed with genozip

Usage: **genols** [options]... [files or directories]...

See also: genozip genounzip genocat

Options:

```
   -q --quiet        Do not show warnings

   -h --help         Show this help page

   -L --license      Show the license terms and conditions for
                     this product

   -V --version      Display version number
```

One or more file or directory names may be given, or if omitted, genols runs on the current directory

Options useful mostly for developers of genozip:

--show-time	Show what functions are consuming the most time
--show-memory	Show what buffers are consuming the most memory
--show-sections	Show the section types of the output genozip file and the compression ratios of each component
--show-alleles	Output allele values to stdout. Each row corresponds to a row in the VCF file. Mixed-ploidy regions are padded, and 2-digit allele values are replaced by an ascii character
--show-dict	Show dictionary fragments written for each variant block (works for genounzip too)
--show-one-dict	<field-name>. Show the dictionary for this field in a tab-separated list - <field-name> may be one of the fields 1-9 (CHROM to FORMAT) or a INFO tag or a FORMAT tag (works for genounzip too)
--show-gt-nodes	Show transposed GT matrix - each value is an index into its dictionary
--show-b250	Show fields 1-9 (CHROM to FORMAT) as well as INFO tags - each value shows the line (counting from 1) and the index into its dictionary (note: REF and ALT are compressed together as they are correlated.) This also works with genounzip, but without the line numbers.
--show-one-b250	<field-name>. Show the values for this field - may be one of the fields 1-9 (CHROM to FORMAT) or an INFO tag
--dump-one-b250	<field-name>. Dump the binary content of this field, exactly as they appear in the genozip format, to stdout - may be one of

	the fields 1-9 (CHROM to FORMAT) or an INFO tag
--show-headers	Show the sections headers (works for genounzip too)
--show-index	Show the content of the random access index
--show-gheader	Show the content of the genozip header (which also includes the list of all sections in the file)
--show-threads	Show thread dispatcher activity
--debug-memory	Buffer allocations and destructions

2. Implementation

genozip operates by segmenting the VCF file into separate sections defined by data type and appropriately processing each section, before applying a general purpose data compressor, bzip2 (Seward, 1996), to each section. genozip executes a number of data transformations that take advantage of data covariance due to linkage disequilibrium, population structure, and potential lab biases, as well as non-textual relationships between numeric values in the file.

First, the VCF file is divided into *variant blocks* of up to 128 MB each (configurable with --vblock), and the samples within each variant block are further divided into *sample blocks* of up to 4,096 samples each (configurable with --sblock), from which the genotypes are extracted and transposed to create a *haplotype matrix*. Prior to compression, each haplotype matrix is further transformed by padding the ploidy to the maximal ploidy represented in the matrix, substituting 2-digit allele values with a single ascii character, and clustering the rows of haplotypes so that similar haplotypes are adjacent to one other. If the --gtshark option is used, clustering is skipped, and GTShark (Deorowicz and Danek, 2019) is used as the final-stage compressor of the *haplotype matrix*, instead of bzip2.

Second, the phase state data (i.e. | or /) are compressed – in the common case where the entire variant block has the same phase state, we drop the phase data entirely and just note the phase state in the variant block header.

Third, the data from each field (CHROM to FORMAT) and subfields of INFO and the sample data (as defined in the FORMAT field) are extracted into separate dictionaries, and their data areis replaced with a dictionary index. An exception is the correlated REF and ALT fields that are combined into one field. For each field, a global dictionary is created for the entire file (or multiple files in case of concatenation), with new values added incrementally as each variant block is parsed, so that only a single pass is needed over the file, and crucially, the compressed file size grows sub-linearly with the number of VCF rows. For the first variant block, the dictionary entries are sorted by frequency, so that the highest frequency entries are efficiently encoded. The dictionaries for each field and the associated index data are then compressed separately. Index data from FORMAT subfields are compressed together as they are often correlated (for example, the DP and AD subfields). Dictionary search is implemented efficiently using hash tables, and an algorithm is run after the analysis of the first variant block to predict their size of the hash table for each field. This algorithm estimates the expected number of unique words of a particular field in the entire file from the gradient of the rate of appearance of new unique words within the first variant block. Extrapolating from the second derivative is obviously error prone, so an algorithm is in place for growing a hash table in run time, if its size was underestimated, while not affecting threads that are concurrently operating on it.

For the non-genotype indexed sample data we apply an additional optimization step of transposing the matrix prior to compressing it, to take advantage of experimental lab bias. In files with a large number of individuals, such as a File1 here, we have observed data differences between individuals that likely result from subtle differences in analysis tools

used – for example, different floating point truncation conventions.

The POS field is often a large contributor to the overall entropy in single or small-sample files. To improve the compression of this field, we compress the difference between successive POS values rather than the POS value itself, thereby reducing the range of values and increasing compressibility.

3. Compression ratio and speed benchmarks in more detail

To benchmark genozip's compression ratio compared to other popular and state-of-the-art compression tools, we used two different files from the 1000 Genome Project (The 1000 Genomes Project Consortium, 2012; Sudmant *et al.*, 2015) that we refer to here as 'File1' and 'File2'. We chose the two files for their substantial difference in their content characteristics (Table S1):

Table S1: Data content of File1 and File2

		**File 1 VCF**			**File 2 VCF**	
Allele values		6.1GB	7.1%		**30.2GB**	**49.2%**
Other sample data		**80.1GB**	**92.3%**		30.2GB	49.2%
Header and columns 1-9		0.5GB	0.6%		0.95GB	1.5%

File1: 1000 Genome Project phase 1 (The 1000 Genomes Project Consortium, 2012; chr1 ftp://ftp-trace.ncbi.nih.gov/1000genomes/ftp/release/20110521/ALL.chr1.phase1_release_v3.20101123.snps_indels_svs.genotypes.vcf.gz). The file contains 1,092 individuals, 3,007,196 variants, and "Other sample data" consisting mostly of the sample fields other than GT, and is the dominant data component in this file.

File2: 1000 Genome Project phase 3 ((Sudmant *et al.*, 2015); chr1 ftp://ftp.ncbi.nlm.nih.gov/1000genomes/ftp/release/20130502/ALL.chr1.phase3_shapeit2_mvncall_integrated_v5a.20130502.genotypes.vcf.gz). The file contains 2,504 individuals, 6,468,347 variants, and Allele values (i.e. the GT subfield) representing ~50% of this file. In this case, "Other sample data" is comprised of the phase state (/ or |) and the tab character that separates the samples – both of which are trivial in terms of compression. Therefore the allele values are about 97% of the remaining data content.

The differences in data content between these two files result in dramatically different compression ratios in all tools. In both cases, though, `genozip` achieves the best compression ratios (Table S2, Figure S1, Figure S2). `genozip` achieves the highest compression ratio amongst the all lossless compression tools, and offers competitive compression even compared to lossy tools such as `GTshark`.

`genozip` was tested in three ways - the first, is its default mode. The second, is with the `--optimize` option which modifies some data in the FORMAT and INFO subfields of VCF file in ways that are typically not significant for analytical purposes, but are quite significant for compression - namely, rounding some floating point numbers to two significant digits and capping some Phred values (see `genozip --help` for a detailed list). This compression by definition is not lossless. As can be seen in Table S2 `--optimize` significantly improves the compression of File1 that consists mainly for FORMAT subfield data, but no has no impact on File2 that has no FORMAT subfield data, The third, is using the `--gtshark` which utilizes `GTShark` for the final stage of compression of the genotype component of the

VCF file, instead of the default `bzlib`. This significantly improves compression in File2 which is enriched in genotype data, but not as much in File1 that consists primarily of FORMAT subfield data.

We faced a number of challenges with the some of other compression tools:

`Hail` failed to decompress because it attempted to create very large intermediate files in the /tmp filesystem. This is a faulty software design as /tmp is typically quite small, and hence decompression of large files is bound to fail due to space constraints as happened in our case. To test a workaround and to allow at least partial inclusion of Hail (Hail Team) in this benchmark despite its malfunctioning, we chose File2 and used Hail's option to shard the decompressed file to many smaller files with its `parallel='separate_header'` option, and then concatenated the file together with the Linux `cat` command. The time shown is the combined to of `Hail` and `cat`.

`bcftools` failed to compress File1 - likely because it is a file created in 2011 prior to the latest versions of `bcftools`.

`GTShark` is not capable of processing FORMAT subfields, and hence is not capable of compressing File1.

`bcftools, Hail` and `GTShark` are not lossless - the decompressed file differs from the original.

Table S2: Compression ratio comparison

Tool	File 1 (MB)	vs. VCF		File 2 (MB)	vs. VCF
**ORIGINAL**	88,775	1X		62,728	
**`genozip`**	**4,430**	**21X**		**257**	**244X**
**`genozip --optimize`**	**3,360**	**26X**		**257**	**244X**
**`genozip --gtshark`**	**4,298**	**21X**		**120**	**523X**
**`gzip`**	10,282	9X		1026	61X
**`bcftools -Ob`**	Incapable	-		1007	62X
**`bgzip`**	10,873	8X		1187	52X
**`bzip2`**	5,767	15X		528	119X
**`xz`**	7,014	13X		367	171X
**`pigz`**	10287	9X		1030	61X
**`gtshark`**	Incapable	-		128	491X
**`Hail`**	18280	4.9X		1258	50X

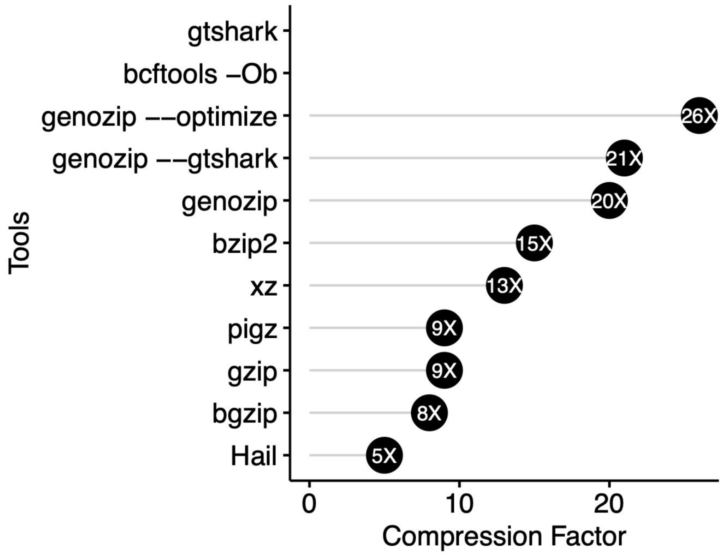

Figure S1: Compression factor for File1

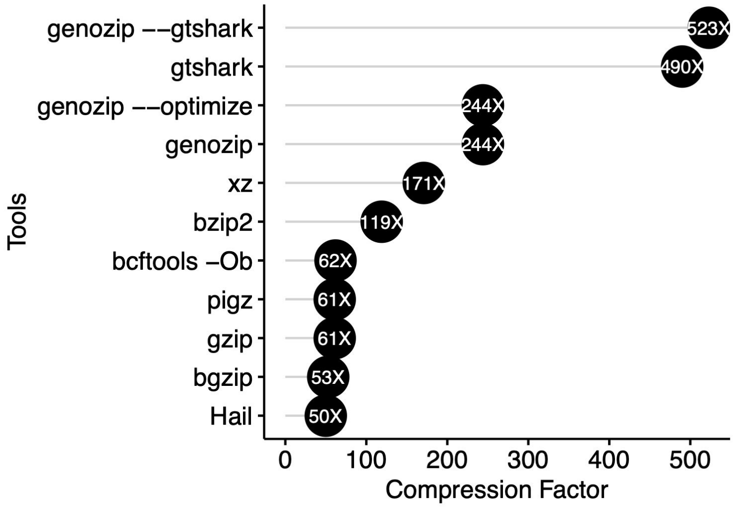

Figure S2: Compression factor for File2

In terms of execution time, genozip is designed to fully leverage the hardware available, unless explicitly restricted by the user. As such, it includes advanced memory and thread management components, that allow almost linear scaling to tens of cores. In table S3, we have the execution time of each tool on our test machine that has 56 physical cores (4 X Intel® Xeon® Gold 6132 CPU @ 2.60GHz) and 755GB of usable memory, running an XFS file system with its default configuration on top of an SSD storage device. While generally multiple users have access to this computer, the benchmark was run one tool at time, and done so at a time when no other users or significant processes were running on the machine.

`bcftools`, `bgzip` and `xz` allow specification on number of threads, and were set to allow them to maximize the utilization of the hardware - "`--threads 56`" for `bgzip` and `bcftools` and "`--threads 0`" for `xz`.

Table S3: Execution time comparison

**Tool**	**Compress**			**Decompress**	
	**File 1**	**File 2**		**File 1**	**File 2**
**`genozip`**	**1’22”**	**1’3”**		**1’56”**	**2’23”**
**`gzip`**	45’19”	10’17”		6’41”	3’47”
**`bcftools`**	N/A	17’27”		N/A	13’8”
**`bgzip`**	1’57”	35”		1’2”	46”
**`bzip2`**	244’30”	207’31”		39’33”	22’9”
**`pigz`**	1’19”	32”		2’58”	1’17”
**`xz`**	21’30”	1’36”		8’26”	2”16
**`gtshark`**	N/A	24’49”		N/A	19’42”
**`Hail`**	4’18”	2’32”[3]		N/A	3’14”

4. Benchmarking of genotype-only compression algorithms

There are a number of algorithms published in recent years focused on compressing genotypes (allele values) within VCF files, while not being capable of compressing arbitrary VCF files. Some are also not capable of decompressing, and all do not guarantee lossless decompression.

Nevertheless, it is interesting to compare the performance of these algorithms on genotype data. In this benchmark we included comparing `genozip` in two modes – its default mode, and with the options `--gtshark -B2048` which would result in the best genotype-data-only compression. We compare against three genotype compression algorithms - `bgt` (Li, 2016), `GTC` (Danek and Deorowicz, 2018) and `GTShark` (Deorowicz and Danek, 2019). We also included the standard tools `gzip` and `bgzip` in this comparison, to appreciate how well all the genotype compression algorithms perform compared to generic compressors

To compare just the genotype data component of a VCF file, we started with File2 from our compression benchmark, and used the `--strip` option of `genocat` to strip out all data, except genotypes, CHROM, POS, REF and ALT fields, and set the FORMAT field to “GT”:

```
genocat ALL.chr1.phase3_shapeit2_mvncall_integrated_v5a.20130502.genotypes.3.
vcf.genozip --strip > file2.stripped.vcf
```

In the results we can see that `genozip` in its default mode results in a better compression ratio of the stripped file than all tools except `GTShark` and `GTC`, while `genozip` with `--gtshark -B2048` is better than any other tool.

Table S4: Compression comparison of a genotype-only file

	Bytes	Compression ratio
Original: `file2.stripped.vcf`	64,956,779,894	
**`genozip`**	**201,369,011**	**323**
**`genozip --gtshark -B2048`**	**60,041,662**	**1082**
`gzip`	851,248,722	76
`bgzip`	939,705,276	69
`bgt`	298,990,428	217
`GTC`	138,182,645	470
`GTShark`	60,297,201	1077

5. Core scalability test - raw data

To test the scalability of genozip with the number of available cores, we ran a compression and decompression test using File1 of our benchmark. We repeated the compression and decompression cycle scaling the number of used cores from 1 to 50 while recording the execution time (see Table S5). We observed that genozip compression scaled approximately linearly up about 28 cores, and then again about linearly up to 50 cores, but with a smaller slope. Decompression, on the other hand, scaled linearly up to about 20 cores after which adding additional cores had no benefit (see Figure 1b). A fundamental constraint on scaling is the need to access the disk file. In the case of genozip, the compressed file is between one and three orders of magnitude smaller than the original file, so it is the original file that is the constraint. We speculate that at least part of the difference in scaling between compression and decompression is the fact that SSD storage is faster in read operations (compression in our case) than write (decompression).

Table S5: execution time in core scalability test

Cores	Compress time (sec)	Uncompress time (sec)	genozip variants/sec	genounzip variants/sec	Cores	Compress time (sec)	Uncompress time (sec)	genozip variants/sec	genounzip variants/sec
1	1,859	1,039	1,618	2,894	26	101	110	29,774	27,338
2	993	551	3,028	5,458	27	100	101	30,072	29,774
3	687	377	4,377	7,977	28	98	118	30,686	25,485
4	520	292	5,783	10,299	29	95	103	31,655	29,196
5	430	239	6,993	12,582	30	95	101	31,655	29,774
6	360	201	8,353	14,961	31	96	120	31,325	25,060
7	320	175	9,397	17,184	32	94	101	31,991	29,774
8	276	159	10,896	18,913	33	93	104	32,335	28,915
9	252	142	11,933	21,177	34	93	103	32,335	29,196
10	228	130	13,189	23,132	35	94	102	31,991	29,482
11	210	119	14,320	25,271	36	94	107	31,991	28,105
12	192	114	15,662	26,379	37	92	103	32,687	29,196
13	179	103	16,800	29,196	38	90	103	33,413	29,196
14	169	98	17,794	30,686	39	93	106	32,335	28,370
15	157	99	19,154	30,376	40	89	103	33,789	29,196
16	149	96	20,183	31,325	41	90	123	33,413	24,449
17	142	96	21,177	31,325	42	91	106	33,046	28,370
18	134	98	22,442	30,686	43	88	104	34,173	28,915
19	130	87	23,132	34,565	44	88	114	34,173	26,379
20	123	93	24,449	32,335	45	91	112	33,046	26,850
21	121	93	24,853	32,335	46	89	110	33,789	27,338
22	115	96	26,150	31,325	47	86	110	34,967	27,338
23	112	108	26,850	27,844	48	86	110	34,967	27,338
24	107	95	28,105	31,655	49	87	107	34,565	28,105
25	104	113	28,915	26,612	50	84	109	35,800	27,589

References

Danek,A. and Deorowicz,S. (2018) GTC: how to maintain huge genotype collections in a compressed form. *Bioinformatics*, 34, 1834–1840.

Deorowicz,S. and Danek,A. (2019) GTShark: genotype compression in large projects. *Bioinformatics*, 35, 4791–4793.

Hail Team Hail.

Li,H. (2016) BGT: efficient and flexible genotype query across many samples. *Bioinformatics*, 32, 590–592.

Seward,J. (1996) bzip2 and libbzip2. *avaliable at http://www. bzip. org*.

Sudmant,P.H. *et al.* (2015) An integrated map of structural variation in 2,504 human genomes. *Nature*, 526, 75–81.

The 1000 Genomes Project Consortium (2012) An integrated map of genetic variation from 1,092 human genomes. *Nature*, 491, 56–65.
